# John Courtenay MacWatters

**Published:** 1938

**Authors:** 


### Obituary

JOHN COURTENAY MacWATTERS, M.B.E., M.R.C.S., L.R.C.P.

We regret to announce the death of Dr. John Courtenay MacWatters at his home, The Golden Beeches, Almondsbury, on 1st February.

He was born in 1873, and educated at Bath Grammar School. Joining the Bristol Medical School in 1891, he qualified in 1896, and following this held several resident appointments at the Bristol Royal Infirmary.

He went into partnership with the late Dr. Irwin at Almondsbury, and, succeeding him, remained there in active practice until his death.

MacWatters was of the best type of general practitioner, enthusiastic and thorough in his work, successfully running, in the midst of a busy practice, a well-equipped bacteriological laboratory, which was not only invaluable in his own work, but in that of others ; he contributed several papers on this subject to the medical press.

During the War he served as M.O. to the following establishments : Almondsbury Hospital ; the Napier Miles Hospital at Kingsweston ; the German Prisoners' Camp at Hallen ; and the Remount Depôt at Shirehampton.

At one time MacWatters was a keen rider to hounds ; this he had to forego owing to the exigencies of practice. His interests were many, from Alpine plants to lathe work, and his many hobbies rounded off a full and successful life.

Despite failing health, during his latter years he never spared himself, as painstaking and genial then as ever, and ready to take the last ounce out of himself for his patient's sake. His loss is one that his relations and friends will feel deeply.

In 1898 he married Miss Bishop, a Sister at the Bristol Royal Infirmary, who predeceased him. He leaves a son and a daughter, to whom we offer our sincerest sympathy.

He had worked in Sir Almroth Wright's laboratory at St. Mary's Hospital, in the early days when it was beginning to attract attention from all over the world. Dr. John Freeman, of St. Mary's, writes of him as he knew him then: "The people working there had more than a laboratory friendship—for every evening they went off and dined together at about eight o'clock and then returned to work in the laboratory, work which usually stopped in the not so very small hours of the morning—any time from 3 to 7 a.m. MacWatters shared this very intimate life and hard work and contributed his full share to the general effort. At the same time he assimilated the views on immunity and resistance which have had such an influence on his subsequent medical career. Unfortunately we were able to see very little of MacWatters once he had settled at Almondsbury—for doctors suffer more than most people in being drowned in their work.

"But when he did reappear in London he was greeted with shouts of delight from his old comrades, whilst he demanded to be regaled with the latest laboratory news and stories, scientific, scandalous, or tall. He was always the cheeriest of companions and for the few short hours that he managed to be with us till his return to work he was the old comrade once again. We had a great affection for him."

